# Influence of Season on the Production of Pneumonia in Children

**Published:** 1843-07-08

**Authors:** 


### INFLUENCE OF SEASON ON THE PRODUCTION OF PNEUMONIA IN CHILDREN.

The season of the year has always been regarded as exercising considerable influence on the development of pneumonia, which has been generally supposed to be most frequent in the later winter and early spring months. The proportion borne by cases of pneumonia to all the cases, 2,450 in number, that came under my notice at the infirmary for children in 1841 and 1842, taking the mean of the two years, is shown to have been, during each three months, as follows:—

| | | |
|---|---|---|
| In 1st three months, | 5.1 | per cent. |
| In 2nd " | 2.5 | " |
| In 3d " | 3.8 | " |
| In 4th " | 5.8 | " |

Although this table does not quite accord with the generally received opinion as to the season when pneumonia is most prevalent, yet it tallies exactly with the results presented in the third report of the Registrar-General. It appears from that document that the greatest mortality from pneumonia among persons under the age of fifteen years takes place in the month of December; and, on a comparison of the different quarters of the year, it will be seen that the deaths from pneumonia are, to the deaths from all causes of persons under fifteen, in the following proportions:—

| | | |
|---|---|---|
| In 1st three months, | 15.3 | per cent. |
| In 2nd " | 11.4 | " |
| In 3d " | 8.9 | " |
| In 4th " | 18.7 | " |

*Dr. West, in Brit. and For. Med. Review.*

